# Patterns of Contraceptive Adoption, Continuation, and Switching after Delivery among Malawian Women

**DOI:** 10.1371/journal.pone.0170284

**Published:** 2017-01-20

**Authors:** Dawn M. Kopp, Nora E. Rosenberg, Gretchen S. Stuart, William C. Miller, Mina C. Hosseinipour, Phylos Bonongwe, Mwawi Mwale, Jennifer H. Tang

**Affiliations:** 1 UNC Project-Malawi, Lilongwe, Malawi; 2 UNC Department of Obstetrics & Gynecology, Chapel Hill, North Carolina, United States of America; 3 UNC Department of Epidemiology, Chapel Hill, North Carolina, United States of America; 4 Division of Epidemiology, College of Public Health, The Ohio State University, Columbus, Ohio, United States of America; 5 UNC Department of Medicine, Chapel Hill, North Carolina, United States of America; 6 Malawi College of Medicine Department of Obstetrics & Gynaecology, Blantyre, Malawi; 7 Bwaila Hospital, Lilongwe District Health Office, Lilongwe, Malawi; London School of Economics and Political Science, UNITED KINGDOM

## Abstract

Women who report use of postpartum family planning may not continue their initial method or use it consistently. Understanding the patterns of method uptake, discontinuation, and switching among women after delivery is important to promote uptake and continuation of effective methods of contraception. This is a secondary analysis of 634 Malawian women enrolled into a prospective cohort study after delivery. They completed baseline surveys upon enrollment and follow-up telephone surveys 3, 6, and 12 months post-delivery. Women were included in this analysis if they had completed at least the 3- and 6-month post-delivery surveys. Descriptive statistics were used to assess contraceptive method mix and patterns of switching, whereas Pearson’s χ^2^ tests were used for bivariable analyses to compare characteristics of women who continued and discontinued their initial post-delivery contraceptive method. Among the 479 women included in this analysis, the use of abstinence/traditional methods decreased and the use of long-acting and permanent methods (LAPM) increased over time. Almost half (47%) discontinued the contraceptive method reported at 3-months post-delivery; women using injectables or LAPM at 3-months post-delivery were significantly more likely to continue their method than those using non-modern methods (p<0.001). Of the 216 women who switched methods, 82% switched to a more or equally effective method. The change in contraceptive method mix and high rate of contraceptive switching in the first 12 months postpartum highlights a need to assist women in accessing effective contraceptives soon after delivery.

## Introduction

Postpartum family planning can help women achieve their fertility goals by allowing them to limit and space their pregnancies. Among nationally representative samples of postpartum women from 21 low- and middle-income countries (including Malawi), 61% had an unmet need for family planning [1]. Of those using family planning, most (51–96%) relied on short-acting methods.

Women who report use of postpartum family planning may not continue the method or use it consistently. Women may start, switch, or discontinue contraceptive methods at various times during the postpartum period. Contraceptive discontinuation and switching may be “active”, as when a woman visits a clinic to have her implant or intrauterine contraception (IUC) removed, or “passive,” as when a pill prescription is not refilled or an appointment for re-injection is missed [2]. In many settings, contraceptive methods that require passive discontinuation (condoms, pills, and injectables) lead to higher rates of discontinuation and pregnancy than those that require active discontinuation (implants, IUC, and sterilization) [3–5]. When women discontinue a method, they may either use no method or switch to another method. Of those that switch, they may switch to a method that is more or less effective at preventing pregnancy.

Understanding the patterns of method uptake, discontinuation, and switching among postpartum women is important to promote uptake and continuation of effective methods of contraception. Therefore, the primary objective of this analysis is to describe the contraceptive method mix at 3, 6, and 12 months post-delivery among a cohort of Malawian women. The secondary objective is to compare characteristics of women who continued and discontinued their initial post-delivery contraceptive method. Finally, we sought to describe patterns of contraceptive switching among women who did not continue their initial post-delivery method.

## Materials and Methods

### Study setting and population

This study involves a secondary analysis of data from a prospective cohort study of postpartum Malawian women (S1 data) [6]. At the beginning of this prospective study, women were recruited from the postpartum unit of Bwaila Hospital, a government district hospital in Lilongwe, Malawi, with over 14,000 deliveries per year.

Ethical approval was obtained from the University of North Carolina School of Medicine Institutional Review Board (IRB) (Approval #13–1084) and the National Health Sciences Research Committee of the Malawi Ministry of Health (Approval #1121). Eligible participants underwent written informed consent.

Eligible women completed an in-person 30-minute baseline survey followed by telephone surveys at 3, 6, and 12 months post-delivery. Criteria for inclusion in the main cohort were as follows: current admission to the postpartum ward at Bwaila Hospital, age 18–45 years, live birth at greater than 28 weeks gestation, fluency in English or Chichewa (the local language), access to a working phone number, and willingness to be contacted by phone for up to one year postpartum. Hormonal and intrauterine contraception were not routinely offered prior to six weeks postpartum at this facility during the study period.

In this current analysis, women were eligible for inclusion if they were recruited into the original cohort, had completed two or more follow-up surveys (at least both the 3-month and 6-month surveys), and were not pregnant at the time of the 6-month survey. Women were determined to be lost to follow-up if they were not able to be reached for a follow-up survey and were not contacted for subsequent surveys. Surveys where women reported pregnancy or had inconsistent or missing contraceptive data were dropped from the analysis.

The main outcomes of interest for this analysis were contraceptive method mix, continuation, and switching. Current contraceptive use was determined by self-report to the question, “Which methods of family planning are you currently using right now?” at each follow-up survey. All methods that were mentioned by the participant were recorded by the interviewer. The contraceptive method mix was classified into 7 categories in ascending order of effectiveness at preventing pregnancy: 1) no method use; 2) abstinence/traditional methods (including breastfeeding, withdrawal, natural family planning); 3) condoms; 4) pills; 5) injectables; 6) long-acting reversible contraception (LARC) (the subdermal contraceptive implant and copper intrauterine device); and 7) female sterilization.[5] Those reporting more than one contraceptive method were classified as using the method most effective at preventing pregnancy.

Women were classified as “continuers” if their response to the current contraceptive method used (including no method used) was the same for all surveys. They were classified as “discontinuers” if they reported different methods on subsequent surveys. Only women who had reported the use of a method other than abstinence at the 3-month or 6-month were included in this analysis, as transition from abstinence to a contraceptive method was not considered discontinuation or continuation.

Method switching was categorized as switching to a less, equally, or more effective contraceptive method using the previously defined order of contraceptive effectiveness. Among method switchers, separate analyses were done on women who ever-used non-modern methods (no method or traditional methods) and women who ever-used modern methods (condoms, pills, injectables, LARC, or sterilization).

### Statistical analysis

Contraceptive patterns were described at each data collection point using frequency analysis. Pearson’s χ^2^ tests were used to compare women included in the analysis to those not included from the original cohort, the method mix at different time points, and characteristics between women who continued their initial contraceptive method and those who did not. Multivariate analysis was not implemented because none of the variables were significantly associated with contraceptive continuation. All data were double entered into a REDCap database, cleaned, merged, exported, and analyzed using Stata Version 13.0 (StataCorp, College Station, TX).

## Results

**Fig 1:**
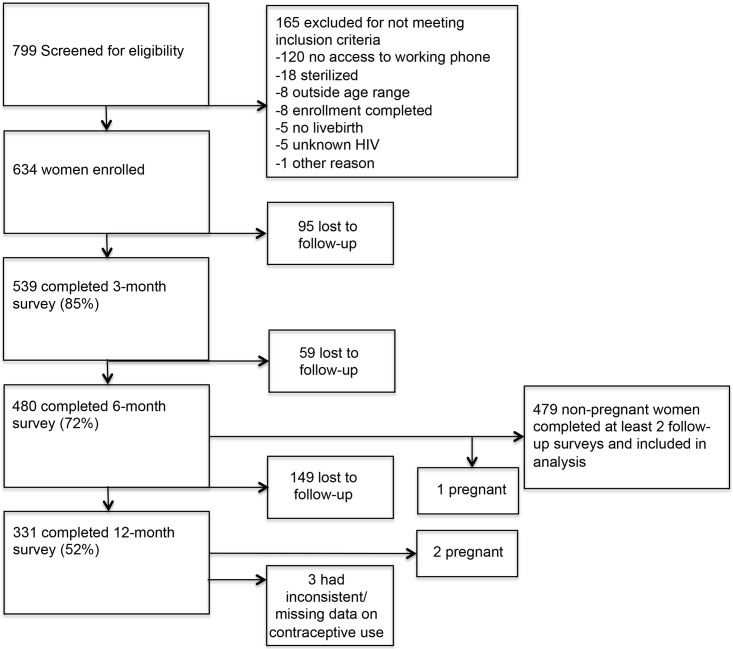
Flow diagram of survey completion.

Overall, 634 women who delivered between May and September 2013 enrolled in the parent study. Of these, 539 women (85%) completed the 3-month survey, 480 women (76%) completed the 6-month survey, and 331 women (52%) completed the 12-month survey (Fig 1). All 480 women who had completed both the 3-month and 6-month surveys were included in the analysis, except for one woman was pregnant at the time of the 6-month survey. Therefore, this analysis includes 479 women. Three women who had inconsistent or missing contraceptive data at the 12-month survey and the data from this survey (but not the 3- or 6-month survey) was censored. Women from the original cohort who were not included in this analysis (because they did not complete two or more follow-up surveys) were younger (p = 0.012), less educated (p<0.001), had more trouble obtaining food, clothing, or medications (p = 0.006), and had fewer living children (p = 0.038) than women who were included.

Nearly all women in the analysis were married, less than 35 years and had completed some primary schooling (Table 1). Most (n = 295, 62%) desired additional children. Most women desired to use LARC at baseline (n = 310, 65%), but only 13% had obtained these by 3 months post-delivery.

**Table 1:** Characteristics of women responding to follow-up surveys, categorized by continuation or discontinuation of their initial contraceptive method reported (n = 479).

	Continued initial method (n = 244)	Discontinued initial method (n = 216)	p-value	Reported abstinence at first two surveys (n = 19)	All women (n = 479)
	n (%)	n (%)		n (%)	n (%)
**Age (years) n (%)**			0.284		
18–24	116 (47.5)	89 (41.2)		9 (47.4)	214 (44.7)
25–34	104 (42.6)	108 (50.0)		9 (47.4)	221 (46.1)
≥35	24 (9.9)	19 (8.8)		1 (5.3)	44 (9.2)
**Relationship status n (%)**			0.338		
Married	235 (96.3)	204 (94.4)		14 (73.7)	453 (94.6)
Unmarried	9 (3.7)	12 (5.6)		5 (26.3)	26 (5.4)
**Education n (%)**			0.341		
None or some primary	70 (28.7)	49 (22.7)		2 (10.5)	121 (25.3)
Primary/some secondary	104 (42.6)	100 (46.3)		9 (47.4)	213 (44.5)
Secondary and beyond	70 (28.7)	67 (31.0)		8 (42.1)	145 (30.3)
**Trouble with food, clothing, or medications n (%)**			0.530		
Yes	124 (50.8)	121 (56.0)		6 (31.6)	251 (52.4)
No	119 (48.8)	94 (43.5)		13 (68.4)	226 (47.2)
Missing	1 (0.4)	1 (0.5)		0 (0.0)	2 (0.4)
**Living children n (%)**			0.805		
1	90 (36.9)	74 (34.3)		13 (68.4)	177 (37.0)
2–3	113 (46.3)	102 (47.2)		4 (21.1)	219 (45.7)
≥4	41 (16.8)	40 (18.5)		2 (10.5)	83 (17.3)
**Desire any more children n (%)**			0.140		
Yes	159 (65.2)	122 (56.5)		14 (73.7)	295 (61.6)
No	80 (32.8)	87 (40.3)		5 (26.3)	172 (36.1)
Don’t know	5 (2.0)	5 (2.3)		0 (0.0)	10 (2.1)
Missing	0 (0.0)	2 (0.9)		0 (0.0)	2 (0.4)
**Most recent pregnancy intention n (%)**			0.380		
Intended	151 (61.9)	125 (57.9)		14 (73.7)	290 (60.5)
Unintended	93 (38.1)	91 (42.1)		5 (26.3)	189 (39.5)
**Most effective method intended at Baseline Survey**			0.753		
Sterilization	37 (15.2)	33 (15.3)		2 (10.5)	72 (15.0)
LARC	150 (61.5)	146 (67.6)		14 (73.7)	310 (64.7)
Injectable	39 (16.0)	25 (11.6)		1 (5.3)	65 (13.6)
Pill	4 (1.6)	4 (1.9)		0 (0.0)	8 (1.7)
Condom	4 (1.6)	3 (1.4)		0 (0.0)	7 (1.5)
Traditional	4 (1.6)	3 (1.4)		2 (10.5)	9 (1.9)
No method	1 (0.4)	0 (0.0)		0 (0.0)	1 (0.2)
Missing	5 (2.1)	2 (0.9)		0 (0.0)	7 (1.5)
**Most effective method initiated at 3-Month Survey**			<0.001		
Sterilization	12 (4.9)	0 (0.0)		N/A	12 (2.5)
LARC	61 (25.0)	4 (1.9)		N/A	65 (13.6)
Injectable	137 (56.1)	44 (20.4)		N/A	181 (37.8)
Pill	12 (4.9)	7 (3.2)		N/A	19 (4.0)
Condom	12 (4.9)	48 (22.2)		N/A	60 (12.5)
Traditional	5 (2.1)	16 (7.4)		N/A	21 (4.4)
No method	5 (2.1)	13 (6.0)		N/A	18 (3.8)
Abstinence	0 (0.0)	84 (38.9)		19 (100.0)	103 (21.5)

**Fig 2:**
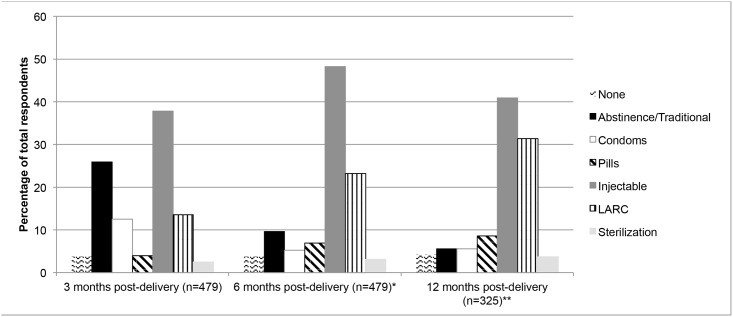
Contraceptive use at each follow-up survey. Among 479 women who completed more than 1 follow-up survey.

The proportion of women using abstinence or traditional methods decreased over time and the proportion of women using long-acting or permanent methods (LAPM, which includes LARC and female sterilization) increased over time (Fig 2). At 3 months post-delivery, the most common method was the injectable (38%), followed by abstinence/traditional methods (26%), LARC (14%), condoms (12%), pills (4%), no method (4%), and female sterilization (2%). At 6 months post-delivery, the most common method was still the injectable (48%), followed by LARC (23%), abstinence/traditional methods (10%), pills (7%), condoms (5%), no method (4%), and female sterilization (3%). At 12 months post-delivery, the most common method remained the injectable (41%), followed by LARC (32%), pills (9%), abstinence/traditional methods (5%), condoms (5%), no method (4%), and female sterilization (4%). The method mix between 3-months and 6-months post-delivery was significantly different, (p<0.001), as was the method mix between 6-months and 12-months post-delivery (p<0.001).

The method mix among a subsample of the 325 women who completed both the 6- and 12- month surveys was examined. At 6 months post-delivery, the most utilized method was injection (45%), followed by LARC (24%), abstinence/traditional methods (10%), pills (8%), condoms (6%), none (4%), and sterilization (3%). At 12 months post-delivery, the most utilized method was injection (41%), followed by LARC (31%), pills (8%), abstinence/traditional methods (6%), condoms (6%), none (4%), and sterilization (4%). Neither the method mix at the 6-month (p = 0.610) or 12-month survey (p = 0.344) was significantly different between those who completed 2 surveys and those that completed all three.

Of the 479 women included in this analysis, 453 (95%) reported attending a postpartum clinic appointment at the time of the 3-month survey. There was no difference in method mix at 3 months (p = 0.362) or 6 months (p = 0.076) post-delivery between women who completed a postpartum appointment when compared to those who did not. However at 12 months post-delivery, the method mix was significantly different (p = 0.002) with those who did not attend a postpartum appointment by 3 months post-delivery to report using no contraceptive method (n = 4, 27%) as frequently as those using injection (n = 4, 27%) and LARC (n = 4, 27%).

Government facilities were the most common place reported for obtainment of condoms (63%), pills (70%), injections (74%), and LARC (72%). However, for sterilization procedures, most women reported receiving this from private facilities (61%).

After excluding women who were abstinent at the 3- and 6-month surveys (n = 19), just over half of women (n = 244/460, 53%) continued using their initial post-delivery method (which could include no method); 216 (47%) discontinued their initial post-delivery method (Table 1). Overall, continuers and discontinuers did not differ significantly in regards to age, parity, marital status, education, or planned contraceptive use. Those who reported LAPM or injectable use at 3 months post-delivery were more likely to continue their method than those using pills, condoms, traditional methods, or no method (p<0.001).

Among the 216 women who did not continue their reported method at 3 months post-delivery, 58% of these switched to a more effective method, 24% switched to a similarly effective contraceptive method, and 18% switched to a less effective method. The most common switch was from condoms to using the injection (n = 27; 13% of switchers).

**Fig 3:**
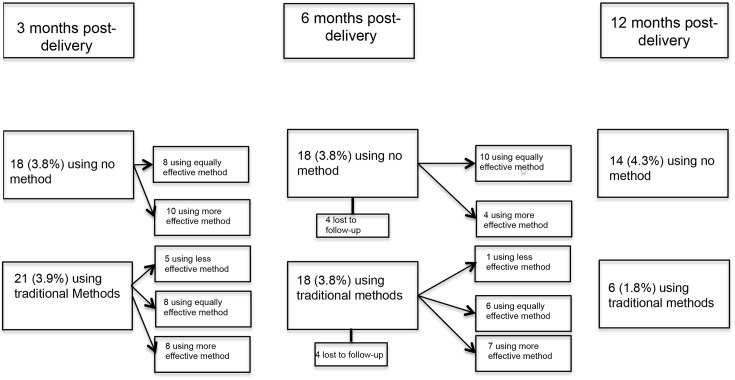
Flow diagram of contraceptive method use and switching among women who ever-used a non-modern method. Where more than one method was used, the method most effective at preventing pregnancy is considered.

**Fig 4:**
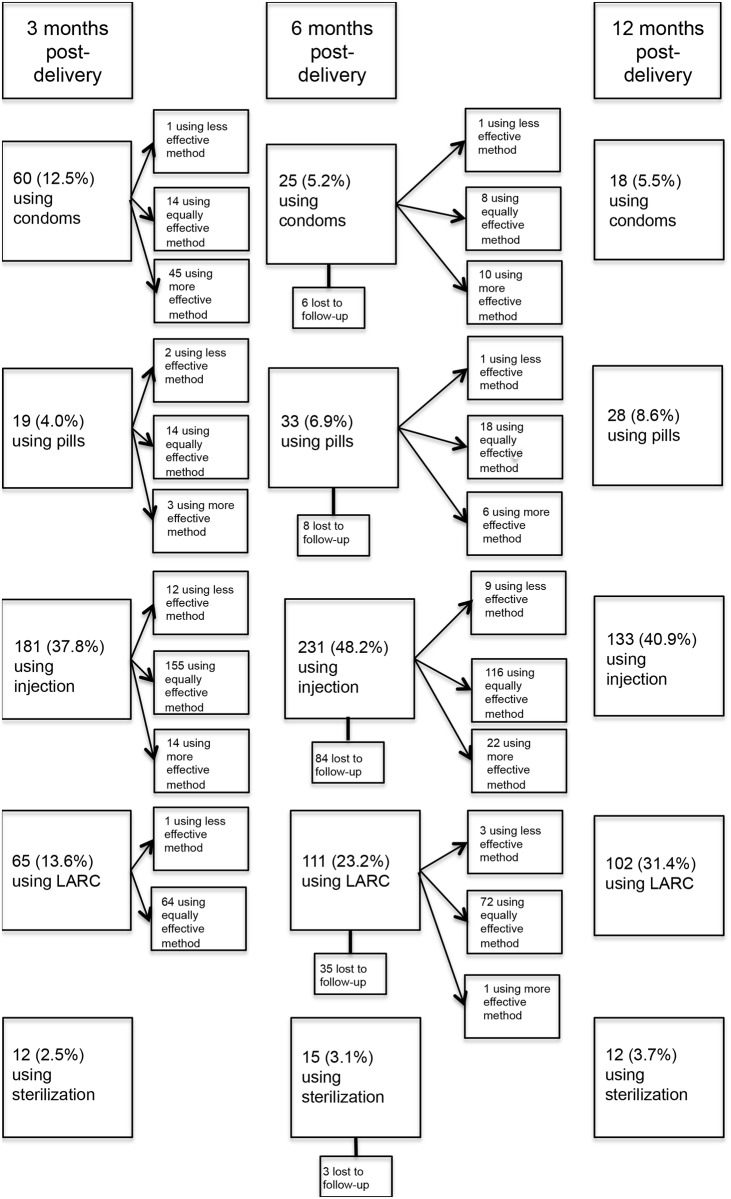
Flow diagram of contraceptive method use and switching among women who ever-used a modern method. Where more than one method was used, the method most effective at preventing pregnancy is considered.

Among those who ever used non-modern methods (Fig 3), the use of traditional methods declined sharply over time: most of these users switched to more effective methods. Among the women using no method at either 3-month or 6-months post-delivery (n = 18), many (50%) continued to use no method, rather than switching to use of a more effective method. Among women using a modern method of contraception at 3-month or 6-months post-delivery (n = 424) (Fig 4), most (93%) reported continuing their method or switching to an equally or more effective method during subsequent surveys.

We also examined women who had ever-used condoms since condom use may be influenced by the perceived risks of both pregnancy and sexually transmitted infections, which may change over time. Among the 107 women who ever-used condoms at 3 or 6 months post-delivery (with or without the use of another contraceptive method), 47 (44%) continued condom use, and 60 (56%) discontinued condom use. Most (n = 40, 67%) condom discontinuers started using a more effective method of contraception when condom use stopped.

## Discussion

In this cohort of postpartum women, the contraceptive method mix changed with more women using LAPM as time from delivery increased. Just over half of women continued the contraceptive method they were using 3 months after delivery. Those that used long-acting methods were more likely to continue using them than those using short-acting methods. Among women who switched their contraceptive method, most switched to a more effective method.

The proportion of women using family planning is higher than in other published studies in Malawi and was found to increase as time from delivery increased. Sexually active women reporting modern contraceptive use increased from 66% at the time of the 3-month survey to 86% at the time of the 6-month survey and 91% at the 12-month survey. Demographic and Health Surveys (DHS) data from Malawi shows that approximately 14% of women are using modern methods of contraception by 3 months after delivery, though this was higher among urban women[7]. The women included in this analysis had high levels of education and were mostly from urban areas in Lilongwe district, which has been shown to have higher postpartum family planning use than most districts in Malawi[8]. The recently published study by Dasgupta used a rural population in northern Malawi and reported that 28.4% of women using modern methods by 6 months and 45.8% using modern methods by 12 months after delivery[9]. However, these proportions included women who reported abstinence and also using a contraceptive method. Between 6–9 months after delivery, 78% of sexually active, menstruating women were using modern methods of contraception, similar to our results.

Other studies in Africa have demonstrated an association with return of menses and uptake of contraception[10]. Unfortunately, ovulation (and return to fertility) occurs prior to the first menstrual cycle, during the period of postpartum amenorrhea[11]. As information on menses was not collected in our surveys, we are unable to comment on this association in our study population.

Changes in method mix after delivery (particularly from less effective to more effective methods) have been described in high-resource settings [12]. In one cohort, American postpartum women initiated pills, patches, or injection earlier than long-acting methods [13]. However, many of these women received family planning methods soon after delivery and method mix at 3, 6, and 12 months post-delivery was largely the same. In another study from the U.S., women commonly switched from barrier methods to a more effective method within 18 months after delivery [14].

Contraceptive continuation and switching in postpartum women has not been studied extensively in low-resource settings. In a study that used a minimum of data from three visits in the first 12 months after delivery in a cohort of urban women in Nairobi, 32% of women discontinued their initial contraceptive method by 6 months and 49% by 12 months after initiation[10], similar to our results. Another recent study of rural postpartum women in Malawi found that only 5 of 169 modern contraceptive users reported use of more than one modern method in the year after delivery[9], though the number of encounters per woman (where an opportunity to record method discontinuation would arise) in this study was not specified. Contraceptive switching at other time points (outside of the postpartum period) has been studied, and a review of DHS data from 25 countries (including Malawi) demonstrated an overall method discontinuation (and switching) rate of 38% by 12 months after initiation[15]. Similar to the findings in this study, lower rates of discontinuation were seen for LAPMs than for condoms, pills, and injection.

The proportion of women who reported condom use at each time point increased over time. However, discontinuation of condom use was high, as 56% of condom users stopped within the study time period. A study of South African women found decreased condom use from 14 weeks post-delivery to 9 months post-delivery among both HIV-infected and HIV-uninfected women [16]. But this study did not assess for uptake of other contraceptive methods, which was demonstrated by a majority of condom discontinuers in our study. This pattern of condom use suggests that some women saw condoms as a temporary method to use until they had secured another desired contraceptive method. Alternatively, it could reflect a decreased concern for risk of sexually transmitted infections over time.

Using participants’ self-report of contraceptive and condom use over a telephone survey may have led to over-reporting of actual use (social desirability bias). The question utilized to elicit contraceptive use may have been leading and contributed to over-reporting. In addition, 95 (15%) of the 634 women from the original cohort never completed any follow-up surveys, and these women may have been more likely to have never initiated a contraceptive method, which would increase our proportions for contraceptive use (selection bias). Another potential limitation of our study is that we only evaluated contraceptive use at three time points and therefore were not able to account for inconsistent use or gaps in contraceptive coverage. Other studies have used contraceptive calendars [2, 10, 17] to collect month-by-month data on contraceptive use or asked participants to report condom and contraceptive use for each sexual act [16]. Additionally, only 69% of the women who completed two follow-up surveys completed the third and final survey at 12 months postpartum. Therefore, our rates of discontinuation and switching may be lower than actual rates if women who were lost-to-follow-up were more likely to discontinue or switch, so our results may not be generalizable to all postpartum women. Further studies should examine if these patterns are similar among postpartum women in other low-resource settings.

In our postpartum population, we observed that modern contraceptive use increased over time and women switched to more effective methods well after the standard six-week postpartum visit. The high rate of contraceptive switching highlights a need to assist women in making these changes without resulting gaps in coverage. The promotion of postpartum contraception needs to encompass a broad time period and focus on easing barriers to accessing effective methods shortly after delivery.

## Supporting Information

S1 Data Supporting information data set. (DTA)
